# Is Team Emotional Composition Essential for Virtual Team Members’ Well-Being? The Role of a Team Emotional Management Intervention

**DOI:** 10.3390/ijerph18094544

**Published:** 2021-04-25

**Authors:** Nuria Gamero, Baltasar González-Anta, Virginia Orengo, Ana Zornoza, Vicente Peñarroja

**Affiliations:** 1 Social Psychology Department, University of Seville, 41004 Sevilla, Spain; 2 Research Institute IDOCAL, University of Valencia, 46010 València, Spain; juan.b.gonzalez@uv.es (B.G.-A.); Virginia.Orengo@uv.es (V.O.); Ana.Zornoza@uv.es (A.Z.); 3 Faculty of Economics and Business, Universitat Oberta de Catalunya, 08018 Barcelona, Spain; vpenarrojac@uoc.edu

**Keywords:** team composition, emotional intelligence, team emotional management, training, members’ well-being, virtual teams

## Abstract

The aim of this study was twofold. First, we examined the relationship between virtual teams’ emotional intelligence composition and three indicators of their members’ well-being, members’ satisfaction with the team, and positive and negative affective states. Second, we analyzed the moderator role of an online team emotional management intervention in the effects of the team emotional intelligence composition. One hundred and two virtual teams participated in an experimental study with repeated measures. Teams were randomly assigned to either an intervention designed to help them detect and manage emotions during virtual teamwork or a control condition (with no intervention). We followed a hierarchical data strategy and examined a number of nested models using Hierarchical Linear Modeling. Our findings showed that virtual teams’ emotional intelligence composition is a key driver of the team members’ well-being, and that a team emotional management intervention moderated the impact of the team composition of emotional intelligence, buffering its influence.

## 1. Introduction

Virtual teams in which employees operate remotely from each other are a reality, and given the numerous technological advancements, they will become even more common in the future [1]. A recent study on virtual teams in 90 countries showed that 89% of the participants worked in virtual teams, and more than half of these teams were composed of members who were geographically dispersed [2]. In virtual teams, members mainly communicate and perform their work using computer-mediated communication [3,4]. Tools such as instant messaging, emails, video calling, or group support systems enable virtual team members to co-ordinate their actions and reach common goals with speed and efficiency [3].

The use of virtual teams enables organizations to do things collectively that face-to-face teams cannot. Nevertheless, due to their characteristics of dispersion and technological mediation, virtual teams have a number of disadvantages or “challenges” that face-to-face teams do not have. Some of these disadvantages are related to the socioemotional management of work teams (e.g., communication and collaboration difficulties, potentially lower team engagement in team members, difficulties in creating trust and shared responsibility, isolation and high levels of social distance) [5]. Virtual team literature indicates that these teams, compared to face-to face teams, “are more oriented toward certain aspects of the task than toward the socio-emotional aspects produced among their members” [6] (p. 83). The lack of attention paid to socioemotional aspects fosters a tendency to behave in a more impersonal, hostile, and uninhibited manner in virtual teams, thus leading to low empathy and personal connection, a lack of clarity, and misunderstandings, distrust, and suspicion [7]. The reduction in emotional verbal information in computer-mediated communication is also a disadvantage. Gilson et al. [8] pointed out that these consequences of working virtually using computer-mediated communication have an impact on members’ well-being. Due to the need to develop positive socioemotional links among team members, a main area of interest for researchers and practitioners is to gain knowledge about how to foster a positive work climate in virtual environments [9]. Researchers have highlighted that developing a positive emotional environment in teams is essential in order to achieve “healthy” teams [10]. Therefore, examining the way emotions are constructed, modified, fostered, or suppressed within virtual teams is an important step in achieving high levels of well-being among the members of virtual teams [8,11]. The majority of the research on well-being has studied it from a hedonic perspective. Thus, well-being is conceptualized as a state of pleasure and an experience of positive affect [12]. Most of the findings indicate that there is a positive relationship between well-being and job performance (see, for instance, [13,14,15,16]). Additionally, team members’ well-being and other outcomes, such as team viability, also help to maintain the group’s performance over time [17]. In a recent review on virtual teams, Gilson et al. [8] stated that an important gap in the virtual team literature involved the absence of an analysis of how to promote high levels of well-being within these kinds of teams.

Kelly and Barsade [18] stated that affective experiences within teams result from individual-level affective factors that team members possess and that define the team’s emotional composition [19,20]. Currently, the role of team composition in terms of emotional skills or traits has been described as an emergent area of team research [10,21], and its impact on members’ well-being has begun to be empirically supported in face-to-face teams (e.g., [22]). Specifically, research has shown that emotional intelligence (i.e., individual emotional skill in detecting, understanding, and managing one’s emotions and those of others; [23]) is a significant predictor of team members’ well-being in face-to-face teams [24]. In computer-mediated work environments, high emotional intelligence among members of virtual teams would also be particularly important because verbal and nonverbal emotional cues are reduced in computer-mediated communication. Thus, virtual teams composed of members with low emotional intelligence may find it more difficult to perceive and manage the members’ emotional experiences within the team [25], negatively influencing members’ well-being. However, to our knowledge, this association has not been examined in virtual contexts. In this regard, we propose that a team emotional management intervention would compensate for this deficit and benefit the team members’ well-being. When we train virtual teams in team emotional management, we are giving their members abilities and tools that will help them to manage the expression of emotions and interactions with their colleagues.

The aim of this study was twofold. First, we analyzed the influence of the team composition of individual emotional intelligence on three indicators of members’ well-being, that is, satisfaction with the team and positive and negative affective states related to virtual work. Second, we examined the moderator role of a team emotional management intervention in the effects of team emotional intelligence composition on virtual teams. We will examine these objectives using a multilevel longitudinal approach.

### 1.1. Team Composition of Emotional Intelligence and Members’ Well-Being

In the past decade, virtual team research has focused on team composition as the critical input for virtual teams’ effectiveness [8]. Most team composition studies have examined members’ gender, race, age, and personality as compositional characteristics [26]. However, the effects of members’ emotional characteristics have mostly been ignored. Virtual teams’ emotional composition is a “bottom-up” component that begins with the variety of individual-level affective components members bring with them to the group interaction.

This virtual team emotional composition would shape the affective experiences of team members [18]. Some researchers have examined trait affect as a compositional variable. Trait affect consists of a predisposition to perceive the world and one’s future positively or negatively [27]. Trait affect permeates our experiences, affecting our state affect and our present and future individual actions [18]. Abundant empirical evidence has shown the effects of trait affect on individual-level behavior. For example, Bagrationi and Thurner [28] showed that employees with a negative view of the future easily feel threatened and tense due to organizational change and show more resistance. In contrast, employees with a positive view of the future are more likely to accept the change and feel greater security, happiness, and enthusiasm about the future situation. Moreover, a positive view of the future has been found to have an influence on team level processes and outcomes [29,30] and team affective states in face-to-face teams. For instance, George [31] found that teams’ mean trait positive and negative affect were related to members’ positive and negative affective states in sales teams. A less studied emotional factor in team emotional composition is individual emotional intelligence [22]. Having emotional intelligence involves being actively able to identify, understand, process, and influence one’s own emotions and those of others in order to guide our feeling, thinking, and action [24]. Emotional intelligence is a personal resource related to positive results because it promotes positive attitudes toward the team task and fosters communication processes and the development of social links among individuals [24]. As with other individual characteristics such as personality and cognitive ability, these individual emotional abilities are aggregated to create a phenomenon at the group level [32], that is, team composition of individual emotional intelligence (EI). Team EI composition refers to the overall level or intensity of EI within the team, and it is typically measured using the mean of members’ scores [33]. Thus, some teams would be composed of members with high levels of EI (high average individual emotional intelligence), and others would be composed of members with low levels of EI (low average individual emotional intelligence).

This team-level emotional characteristic may produce top-down influences on the affective states of virtual team members through its effect on patterns of interaction and social influence among members [33]. Forsythe [34] suggested the importance of having not only individual task-related knowledge, skills, and abilities when assembling teams, but also social and emotional skills that influence conscious affective sharing of emotions, which then promotes team-level functioning. In agreement with this author, different team composition models indicate that overall team functioning is improved by selecting individuals with high levels of specific skills. For instance, traditional personnel-position fit models, such as Muchinsky and Monahan’s person-environment model of supplementary fit [35], argue that all virtual team members should have high levels of socio-emotional skills in order to facilitate interpersonal interactions. Personnel models with teamwork considerations assume that when members, on average, possess greater team-related competencies, the team is more likely to function effectively [36]. Finally, team profile models suggest that some distributions or more complex profiles of members’ attributes, including task- and socioemotional-related attributes, contribute to the ability to orchestrate teamwork functions [37].

Empirical evidence has shown that, in face-to-face teams, EI is positively related to the quality and effectiveness of interpersonal interactions [38]. For instance, Jordan and Troth [39] and Offerman et al. [40] found that teams with higher levels of EI functioned better than teams with lower levels of EI. Scholars have also found that teams with members with high EI reported higher levels of intra-team trust and psychological safety among team members and lower levels of conflict, greater team learning, and more collaborative decision-making [41,42,43]. Depending on a team’s EI composition, group functioning will be modified, and affective outcomes may differ.

Transferring these findings to virtual teams, virtual teams composed of members with higher emotional abilities will be more effective at detecting and managing emotions [44], which could be reduced in computer-mediated communication environments [45]. Different approaches argue that, in a computer-mediated communication context, it is difficult to detect and therefore manage members’ emotions. For instance, cues-filtered-out approaches [25,46] attribute the lack of emotions in virtual teams to the absence of non-verbal and non-textual cues in virtual environments. For some researchers, the restrictions on transmitting cues and deindividuation processes cause socio-emotional information to be reduced, if not completely lost (e.g., [47]). However, Social Information Processing Theory [48] argues that, in computer-mediated communication, it is possible to convey affective and emotional information and relational communication, despite the reduced availability of nonverbal cues. This theory states that members are able to exchange socio-emotional information as if they were in face-to-face settings, but this requires exchanging several messages and adapting the content and style of written messages online [49]. Thus, virtual teams need more time to process socio-emotional information and develop effective socioemotional relationships among team members, compared to face-to-face teams. Therefore, virtual teams whose members have better emotional awareness and emotional management abilities will be more effective in developing accurate collective emotional knowledge, attitudes, and behaviors over time that facilitate the management of the interpersonal relationships [50]. Hence, in virtual teams with a high average level of EI, interpersonal interactions and group functioning are better than in teams with low levels of EI. These team EI levels, in turn, influence team members’ emotional states and satisfaction with the team.

Despite prior research on the consequences of team composition in virtual teams [8], to our knowledge, no study has analyzed the influence of team EI composition on virtual team processes or outcomes. Considering these theoretical arguments, we formulate the following hypotheses:

**Hypothesis** **1.** *Team EI composition will be positively associated with members’ satisfaction with the team (Hypothesis 1a) positive affective states (Hypothesis 1b) and negatively associated with members’ negative affective states (Hypothesis 1c) over time.*

### 1.2. The Moderator Role of a Virtual Team Emotional Management Intervention

Based on the above, we can conclude that it is important to design teams whose members have high EI in order to foster members’ well-being within virtual teams [51,52]. Nevertheless, this might not be an easy task. Virtual teams are often constructed because organizations require skills, local knowledge, experience, resources, or expertise from employees who are geographically distributed [5]. Organizations can also face particular difficulties in selecting team members who have the balance of technical and interpersonal skills and abilities required to work virtually. Moreover, this can be especially complex when virtual teams are composed of employees who come from different organizations [33,53], or in teams with a short time frame, such as virtual project teams.

The drawbacks of virtual teams composed of members with low EI could be overcome by specifically training these teams to develop team competencies to manage emotions. Team emotional management refers to a team’s overall ability to manage members’ emotions and interpersonal relationships [54]. Team emotional management is crucial for developing a positive team atmosphere [51]. Thus, this overall ability to manage emotions influences expressions and displays of emotional cues [55]. Team members’ displays of emotions may serve as behavioral cues used by other members to evaluate their immediate environment and synchronize their own emotional displays [56]. Teams with high levels of team emotional management may be better at stimulating, displaying, and maintaining positive emotions and building a positive team climate [54]. Thus, we propose a team emotional management (TEM) intervention to enhance team competencies in expressing, recognizing, and managing emotions, and develop a positive climate that can minimize the negative impact of a team composed of members with different levels of EI. The aim of a TEM intervention is to improve the team’s ability to effectively manage emotions while interacting in a computer-mediated communication context [21,54]. As Beranek and collaborators [57,58] suggested, the training the team receives in developing relational links is critical. Learning competencies that allow effective emotional communication and the management of emotional experiences within virtual teams could mitigate the constrictions initially attributed to virtual teamwork.

A TEM intervention for virtual teams would be especially important for teams whose interactions exclusively take place through written communication. In online written communication, the communication medium used by our virtual teams, the lack of vocal cues means that socio-emotional information has to be transmitted through written messages. Thus, in order to manage emotions, virtual team members need to be competent in using verbal resources such as paralinguistic cues, management of timing, and emoticons [59,60]. All these cues play a key role in online communication [61]. Emotional information can be transmitted and managed in online written communication using paralinguistic cues, which can also assist with impression formation [59]. Typographical marks, such as exclamation points, ellipses, and the use of capital letters, add emotional meaning to written messages (e.g., excitement, trailing thoughts, and shouting, respectively) [62]. Emoticons help to clarify, strengthen, or soften the meaning of a written message as an alternative to facial cues found in face-to-face contexts. Thus, emoticons can serve as markers of a positive attitude, providing information about how a written message is supposed to be interpreted (e.g., following messages that are intended to be humorous). They also strengthen and intensify positive expressions (such as greetings, thanks, compliments, etc.) and temper messages with negative content (such as requests, corrections, rejections, and complaints, etc.) [60]. The use of time, known as chronemics, is another nonverbal cue used when forming impressions in online written communication [62]. The timing of sending and receiving messages (such as pauses or silence; [61]) and the frequency and duration of online interactions [63] influence impression development and provide information that can be interpreted in a variety of ways. For example, an immediate response implies priority and importance [62], whereas if a receiver waits a long time to respond, a message can be interpreted as less credible [61] or indicate superiority of the receiver or a perceived lack of status of the sender [62].

Thus, an online intervention designed to facilitate the use of socio-emotional cues to improve affect management in online written communication would mitigate the impact of team EI composition [58]. Thus, we formulated the following hypotheses:

**Hypothesis** **2.** *A TEM intervention will moderate the relationship between team EI composition and members’ satisfaction with the team (Hypothesis 2a), positive affective states (Hypothesis 2b), and negative affective states (Hypothesis 2c), so that the influence of team EI composition will be weaker in teams that receive the intervention.*

## 2. Materials and Methods

### 2.1. Sample

The sample was composed of 101 virtual teams with four members each and one virtual team with three members (407 participants). All the participants were undergraduate psychology and labor relations students from two public Spanish universities. Based on Bello and colleagues [64], the use of a student sample is justified when a study is experimental or longitudinal in nature, is guided by a well-defined theory, and makes sophisticated predictions that are confirmed by the results. In this case, it is likely that the results can be generalized to the target population. Moreover, as Gilson and colleagues pointed out [8], much of the research on virtual teams is conducted with students as participants. Our virtual teams composed of students also met Kozlowski and Bell’s [31] criteria for defining a work team in an organizational setting. First, members of each team share common goals and work processes. Second, the functional relationships among team members and their nearness promote team members’ social interaction. Third, team members exhibit task interdependence. Finally, team members must coordinate with each other to carry out their tasks. Participants took part in the experiment voluntarily and received course credit for their collaboration. The average age of this study sample was 20.8 years (*SD* = 4.6), and 75.4% of the participants were women. Regarding educational level, 83% had completed secondary education.

### 2.2. Design and Procedure

We conducted an experiment using a mixed factorial design with a between-subjects condition (with vs. without intervention) and a within-subjects condition (measurement time: Sessions I-III) to test the study hypotheses. This experiment received formal approval from the institutional Ethics Research Committee of the University. We presented the experiment as a study on innovative teaching practices that would allow participants to learn about working in virtual teams. Hence, participants were not aware of the purpose of the study until the debriefing meeting. Students who were interested in participating in the study attended an informative meeting. In this meeting, they received instructions about the procedure and agreed to participate in the experiment. They signed a written consent for their participation, and they were assured that they could not be identified via this study because we fully anonymized their individual responses.

We randomly assigned participants to one of the 102 virtual teams. All the virtual teams in the study maintained the composition of their members during the entire experiment. Next, we randomly allocated each team to the experimental (teams with intervention) or control condition (without intervention).

Virtual teams had to perform intellective tasks in three work sessions. We adapted three intellective decision-making tasks, following the task model of McGrath [65], which are also called “survival” tasks. They were integrated in a common narrative during the entire experiment through digital storytelling. The three tasks were The Bushfire Survival Situation, a survival in a bunker situation, and Lost in The Desert [66,67]. In each task, team members receive a list of items and objects they have to put in order depending on their usefulness for their survival. To solve the task, participants first made the list individually, and then as a team. They had to combine individual ideas and interact with each other via the written chat in the virtual collaborative space. These tasks have a definitive solution provided by experts as an objective result with which to compare individual and team rankings. These types of tasks are commonly used in experimental studies on teamwork, and they have been found to be very useful for team development [68].

Teamwork carried out during the experimental sessions took place in a virtual collaborative space online in real time. This electronic platform was specifically designed to combine different features of other well-known collaborative tools. Participants received instructions, activities, questionnaires, and messages in this virtual space. They also worked collaboratively on shared documents, and there was a synchronous chat to communicate with the rest of the team members. Participants received guidance for using the chat in order to meet the requirements of the experimental procedure. Therefore, they did not know the identities of the other members, and they were asked to avoid disclosing personal information while participating in the experimental sessions. A pilot study was carried out to test the time required to solve the tasks and the tools used on the software platform.

**Figure 1:**
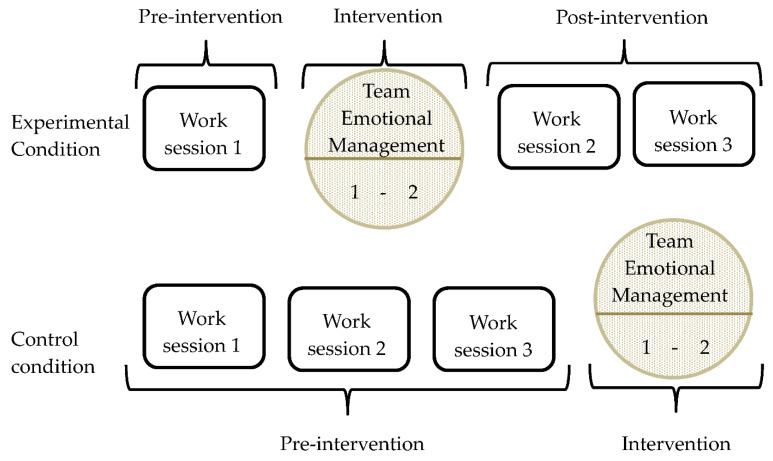
Experimental design of the study.

Virtual teams allocated to the experimental condition (teams with the intervention) received our team emotional management intervention after the first work session. Virtual teams allocated to the control condition (teams without the intervention) received our intervention after the teams had finished the three work sessions (see Figure 1).

We administered the Team Emotional Management (TEM) intervention in two training phases in the virtual collaborative space. The first training phase was designed to teach participants how to identify, express, and regulate emotions in virtual teams. We first discussed the importance of socio-emotional factors in virtual teams and work contexts. We taught them about written communication and the visibility of emotions online, the perception of anonymity among participants, and how participants in virtual environments had to construct shared code systems to avoid misunderstandings. Second, we taught them about resources to explicitly express emotions in virtual environments. In this section, we explained the use of paralinguistic cues (abuse, use of idioms and onomatopoeia, words taken from different languages); the management of temporal resources and chronemic cues (silences, the length of pauses between interactions and interpretation of delays); and the use of graphic symbols and emoticons (e.g., ¯\_(ツ)_/¯) to substitute for a message or complete sentence or complement it. These resources were explained by focusing on their role in improving (or deteriorating) the expression and regulation of emotions in virtual environments, due to their impact on the interaction process. Third, we explained what emotional regulation is, and we taught them strategies to implement in virtual environments (e.g., using humor in tense group situations, addressing others by name to feel closer, managing response times when a negative situation is experienced in the team, or re-evaluating a situation, looking for more positive ways to reinterpret a message, etc.) [59,60].

The second TEM intervention training phase focused on managing the emotional climate while working online as a team. We introduced the work session by explaining the importance of a good emotional climate in virtual teams for team performance and other team processes and outcomes. Next, we gave them five strategies to manage emotional climate while participating in a virtual team: (1) Acknowledge the effort and contribution of the members, (2) send messages to motivate the team to achieve common goals, (3) reward collective effort and success through written messages, (4) use jokes and humor while working to boost positive feelings, and (5) re-evaluate the situation.

Each phase had two parts. The first part was completed individually and consisted of a brief video-training and practical activities. After all the members of a team had completed this part, in the second part, team members performed a set of group activities that were similar to the previous ones and related to the contents of the individual part. However, they now had to agree on the answers in order to create a common frame of reference for the use of the resources and strategies learnt (e.g., write an example of how you will use emoticons to intensify or soften written messages).

A battery of questionnaires examining the variables of our study was administered at the same points in both conditions. Team EI composition was measured during the informative meeting with participants before the pre-intervention. Satisfaction with the team, and positive and negative affective states, were measured at the end of Session 1 and Session 3.

### 2.3. Measures

Team EI composition. This variable was measured by Carvalho et al. [69] with an adapted version of the Wong Law Emotional Intelligence Scale (WLEIS) Scale. The scale consisted of 16 items rated on a Likert scale ranging from one (strongly disagree) to seven (strongly agree). An example item is “I am sensitive to the feelings and emotions of others”. Cronbach’s α for this variable was 0.82. Moreover, although measured at the individual level, EI was operationalized at the team level using an additive composition model (team average; [70]).

Satisfaction with the team. This variable was measured with seven items taken from Medina’s scale [71], rated on a five-point Likert scale ranging from one (strongly disagree) to five (strongly agree). A sample item is “On the whole, I am satisfied with the team”. Cronbach’s α was 0.94, and 0.97 for Sessions 1 and 3, respectively.

Positive affective states. This variable was measured by six items adapted from Segura and González-Roma [72]. The items were rated on a five-point Likert scale ranging from one (not at all) to five (entirely). An example of an item is “This task made me feel happy”. Cronbach’s α was 0.86 and 0.91 for sessions 1 and 3, respectively.

Negative affective states. This variable was also measured with six items adapted from Segura and González-Roma [72]. As on the previous scale, the items were rated on a five-point Likert scale ranging from one (not at all) to five (entirely). An example of an item is “This task made me feel tense”. Cronbach’s α was 0.69 and 0.77 for Sessions 1 and 3, respectively.

Given that positive and negative affective states and individual satisfaction with the team were measured simultaneously in Sessions 1 and 3, we conducted a confirmatory factor analysis to ascertain whether items used in the study measured three discriminable factors. The analysis was conducted separately for Session 1 and Session 3. The item covariance matrix was the input matrix, and the model parameters were estimated by means of maximum likelihood methods. Considering that the item distributions departed from normality, we computed the chi-square fit statistic corrected for non-normality. The hypothesized three-factor model showed an acceptable fit to data in Session 1 (*X*^2^/*df* = 3.25, *p* < 0.01; RMSEA = 0.07; CFI = 0.92; TLI = 0.91; SRMR = 0.10) and Session 3 (*X*^2^/*df* = 2.34, *p* < 0.01; RMSEA = 0.06; CFI = 0.96; TLI = 0.95; SRMR = 0.06). We compared the fit of the three-factor model with the fit of an alternative one-factor model that posited that the three dependent variables were not discriminable. The fit of the one-factor model was not adequate in Session 1 (*X*^2^/*df* = 8.45, *p* < 0.01; RMSEA = 0.13; CFI = 0.75; TLI = 0.70; SRMR = 0.13) or Session 3 (*X*^2^/*df* = 10.17, *p* < 0.01; RMSEA = 0.15; CFI = 0.71; TLI = 0.67; SRMR = 0.14). The Satorra Bentler chi-square difference test (TRd) is significant when both models are compared in both sessions (TRd in Session 1= 196.82, Δdf = 3, *p* < 0.01 and TRd in Session 3= 368.30, Δdf = 4, *p* < 0.01), providing support for the three-factor model. These results confirmed that the items in our dependent variables measured three discriminable factors.

Control variables. In this study, we controlled the stability effect of our dependent variables (that is, the dependent variable measured in Session 1). Additionally, we controlled the composition of the virtual teams with regard to two variables, university of origin and participants’ personal interests when carrying out the “survival” tasks used in the experiment. Information about their interests was obtained through a questionnaire completed in the informative meeting that measured two types of survival roles, impulsive or reflexive. Team characteristic was a dichotomous variable indicating the homogeneity of the participants in each team regarding the university of origin and the personal interests of the members. Thus, on the one hand, teams whose members all came from the same university and had similar personal interests, based on the bogus questionnaire, received a score of 0. On the other hand, teams with two members from each university and different personal interests received a score of 1.

Experimental manipulation check scale. We checked the correct experimental manipulation of the team intervention using a 10-item scale with questions about the contents of the intervention. A sample item is “We used paralanguage signs (e.g., onomatopoeia, capital letters) to qualify our written messages”. Responses were given on a four-point Likert scale ranging from 1 (not at all) to 4 (very much). Cronbach’s α for this scale was 0.89 for Session 3. This scale was also aggregated at the team level. The mean of the *r_WG_*_(J)_ was 0.82 (*SD* = 0.15) for Session 3. ICC(1) was 0.33. Thus, we proceeded to aggregate the data at the team level.

## 3. Results

### 3.1. Manipulation Check

Using the experimental manipulation check scale, we compared the means in the experimental and control conditions on the use of TEM resources and strategies after interacting together in the work sessions. Results showed that this intervention had the expected effect. Participants’ means in the experimental condition indicated that they used emotional management strategies with their respective teammates more often than participants in the control condition, after receiving the TEM intervention in Session 3 (intervention: *M* = 3.0; *SD* = 0.44; control condition: *M* = 2.7; *SD* = 0.43; t_(100)_ = −3.42; *p* < 0.001).

### 3.2. Preliminary Results

Means, standard deviations, and bivariate zero-order correlations are presented in Table 1 (control condition; CC) and Table 2 (experimental condition; EC). Team EI composition was positively related to satisfaction with the team (*r*_CC_ = 0.27, *p* < 0.01; *r*_EC_ = 0.28, *p* < 0.01) and positive affective states (*r*_CC_ = 0.15, *p* < 0.05; *r*_EC_ = 0.17, *p* < 0.05) in Session 3 in both conditions. However, this variable was only significantly related to negative affective states (*r*_CC_ = 0.30, *p* < 0.01; *r*_EC_ = 0.06, *n.s*.) measured in Session 3 in the control condition.

The team characteristics variable was significantly related to satisfaction with the team (*r*_EC_ = 0.28, *p* < 0.01) and positive affective states in Session 3 (*r*_EC_ = 0.20, *p* < 0.01) in the experimental condition, supporting the need to control this variable. Moreover, although the students represented a homogeneous group in terms of level of education, we carried out an ad hoc analysis and tested whether participants’ gender and age could be affecting our dependent variables. Thus, Box’s M statistic was calculated for satisfaction with the team and positive and negative affective states [73,74]. This index allowed us to check whether our data could be combined and analyzed together, considering differences in participants’ gender and age. Box’s M statistic tests the null hypothesis, according to which the covariance matrix between the study variables is the same across gender and age. In the case of non-significance, it is possible to jointly analyze the data. Regarding gender, the results were as follows: M = 0.08 (*p* = 0.78) for satisfaction with the team; M = 2.78 (*p* = 0.10) for positive affective states; and M = 0.03 (*p* = 0.85) for negative affective states. Regarding participants’ age, the results were as follows: M = 5.85 (*p* = 0.06) for satisfaction with the team; M = 0.05 (*p* = 0.97) for positive affective states; and M = 0.69 (*p* = 0.71) for negative affective states. Therefore, data gathered from participants with different genders and ages were combined and analyzed together, given that neither of these variables influenced our dependent variables.

### 3.3. Hypothesis Testing

To test Hypothesis 1, we followed a hierarchical data strategy and examined three nested models using Multigroup Hierarchical Linear Modelling: Null model, Model 1, and Model 2. Prior to testing the hypotheses, it is necessary to determine that there is sufficient variance in the criterion variables at all levels of analysis [75]. Thus, a null model was tested, including the intercept as the only predictor. The null model determined the proportion of variance in satisfaction with the team and positive and negative affective states at Level 1 (within teams) and Level 2 (between teams). The intraclass correlation (ICC) was computed in this model. In Model 1, the control variables (stability effect of the dependent variable and the team characteristics variable) were entered. In Model 2, team EI composition was introduced. We examined the models in the control condition (teams without intervention) and investigated whether there was significant variance in the intercepts and slopes across virtual teams to specify the best fitting random coefficient model.

To test Hypothesis 2, we examined the same nested models in the experimental condition (teams with intervention), and we compared the results for the two conditions.

#### 3.3.1. Satisfaction with the Team as Dependent Variable. Hypothesis 1a and 2a

Regarding the null model, the ICC (1) values were significant in both the control and experimental conditions for satisfaction with the team (ICC (1) = 0.44 and 0.42, respectively), showing that team membership explained a sufficient proportion of variance in this criterion variable.

Hypothesis 1a proposed that team EI composition would influence satisfaction with the team (see Table 3). Considering the control condition, when control variables were entered (Model 1), results revealed that Model 1 showed a significant improvement over the null model (difference of −2 * log = 5.54; *df* = 2; *p* < 0.01). Satisfaction with the team measured in Session 1 was positively associated with satisfaction with the team in Session 3 (*β* = 0.23, *p* < 0.01). When team EI composition was entered (Model 2), findings revealed that Model 2 showed a significant improvement over Model 1 (difference of −2 * log = 3.52; *df* = 1; *p* < 0.05). Team EI composition was positively associated with satisfaction with the team in Session 3 (*β* = 0.50, *p* < 0.05). Therefore, Hypothesis 1a was supported. 

Hypothesis 2a proposed that the influence of team EI composition on satisfaction with the team would be moderated by the TEM intervention. Thus, we tested the same models in the experimental condition. Model 1 showed a significant improvement over the null model (difference of −2 * log = 27.85; *df* = 2; *p* < 0.01). Satisfaction with the team measured in Session 1 was positively associated with satisfaction with the team at Time 3 (β = 0.42, *p* < 0.01). Model 2 showed a non-significant improvement over Model 1 (difference of −2 * log = 1.87; *df* = 1; *n.s*.). Team EI composition was not significantly associated with satisfaction with the team in Session 3 (*β* = 0.36, *n.s*.). Our findings showed that the effect of team EI composition on satisfaction with the team in Model 2 was significant in the no-intervention condition (*β* = 0.50, *p* < 0.05) and non-significant in the intervention condition (*β* = 0.36, *n.s*.). Therefore, Hypothesis 2a was supported.

#### 3.3.2. Positive Affective States as Dependent Variable. Hypothesis 1b and 2b

Regarding the null model, the ICC(1) values were significant for positive affective state in both the control and experimental conditions (ICC(1) = 0.35 and 0.34, respectively), showing that team membership explained a sufficient proportion of variance in this criterion variable.

Hypothesis 1b proposed that team EI composition would influence positive affective states (see Table 4). Considering the control condition, when the control variables were entered (Model 1), the results revealed that Model 1 showed a significant improvement over the null model (difference of −2 * log = 3.54; *df* = 2; *p* < 0.05). Positive affective states measured in Session 1 were positively associated with positive affective states in Session 3 (*β* = 0.28, *p* < 0.01). When team EI composition was introduced (Model 2), our findings revealed that Model 2 showed a non-significant improvement over Model 1 (difference of −2 * log = 0.98; *df* = 1; *n.s*.). Team EI composition was not significantly associated with positive affective states in Session 3 (*β* = 0.30, *n.s*.). Therefore, Hypothesis 1b was not supported.

Hypothesis 2b proposed that the influence of team EI composition on positive affective states would be moderated by the TEM intervention. Thus, we tested the same models in the experimental condition. Model 1 showed a significant improvement over the null model (difference of −2 * log = 39.43; *df* = 2; *p* < 0.01). Positive affective states measured in Session 1 were positively associated with positive affective states in Session 3 (*β* = 0.44, *p* < 0.01). Model 2 showed a non-significant improvement over Model 1 (difference of −2 * log = 0.44; *df* = 1; *n.s*.). Team EI composition was not significantly associated with positive affective states at Time 3 (*β* = 0.21, *n.s*.). Our findings showed that the effects of team EI composition on positive affective states was non-significant in both conditions. Therefore, Hypothesis 2b was not supported.

#### 3.3.3. Negative Affective States as Dependent Variable. Hypothesis 1c and 2c

Regarding the null model, the ICC(1) values were for negative affective state were significant in both the control and experimental conditions (ICC(1) = 0.30 and 0.31, respectively), showing that team membership explained a sufficient proportion of variance in this criterion variable.

Hypothesis 1c proposed that team EI composition would influence negative affective states (see Table 5). Considering the control condition, when control variables were entered (Model 1), the results revealed that Model 1 did not show a significant improvement over the null model (difference of −2 * log = 2.95; *df* = 2; *n.s.*). However, negative affective states measured in Session 1 were positively associated with negative affective states in Session 3 (*β* = 0.18, *p* < 0.01). When team EI composition was introduced (Model 2), our findings revealed that Model 2 showed a significant improvement over Model 1 (difference of −2 * log = 12.91; *df* = 1; *p* < 0.01). Team EI composition was negatively associated with negative affective states at Time 3 (*β* = −0.69, *p* < 0.01). Therefore, Hypothesis 1c was supported.

Hypothesis 2c proposed that the influence of team EI composition on negative affective states would be moderated by the TEM intervention. Thus, we tested the same models in the experimental condition. Model 1 did not show a significant improvement over the null model (difference of −2 * log = −4.62; *df* = 2; *n.s*.). Negative affective states measured in Session 1 were not significantly associated with negative affective states in Session 3 (*β* = 0.04, *n.s*.). Model 2 showed a non-significant improvement over Model 1 (difference of −2 * log = −1.47; *df* = 1; *n.s*.). Team EI composition was not significantly associated with negative affective states at Time 3 (*β* = −0.06, *n.s*.). Our findings showed that the effect of team EI composition on negative affective states, tested in Model 2, was significant in the control groups (*β* = −0.69, *p* < 0.01) and non-significant in the experimental groups (*β* = −0.06, *n.s*.). Therefore, Hypothesis 2c was supported.

## 4. Discussion

The purpose of our study was twofold. First, we investigated the extent to which team EI composition predicted members’ satisfaction with the team and affective states in virtual teams. Second, we examined whether a TEM intervention had an impact on these relationships, buffering the effects of team EI composition on individual well-being indicators.

We hypothesized that teams’ emotional intelligence composition would influence virtual team members’ satisfaction and affective states over the team’s lifespan. Few studies have focused on the effects of team emotional intelligence composition on individual and team results (e.g., [31]), and to our knowledge, no study has examined the influence of individual emotional intelligence as a team composition characteristic in virtual teams. We found empirical support for the relationship between team EI composition and members’ well-being over time. According to Social Information Processing Theory [48], due to the adaptation of the content and style of messages in online written communication, virtual teams require a longer time span to process and manage collective emotional knowledge and behaviors and develop successful interpersonal relations [49]. Our findings showed that team EI composition contributes to processing socioemotional information and managing interpersonal links over time. Our results also support theoretical models of team composition effects. These models state that when members, on average, have greater team-related competencies, the team is more likely to function effectively because these competencies facilitate interpersonal interactions [33,35,37]. Nevertheless, team EI composition influenced the change in satisfaction with the team and negative affective states over time, but it did not influence positive affective states. It is possible to find an explanation for these results. Compared to face-to-face teams, virtual team members behave in a more uninhibited, impersonal, and even hostile manner [49]. Because members with high levels of emotional intelligence are probably attuned to their own emotions and the emotions of others, they are able to detect these negative emotions and manage them, in order to enhance the quality of the communication among the members and reduce potential conflicts within the virtual team. Moreover, the lack of negative emotional expressions within virtual teams may be interpreted as a sign of suitable team functioning [25], increasing, in turn, members’ satisfaction with the team. Nonetheless, team EI composition did not significantly promote work-related positive states within the virtual team. We expected that individual positive affective states would increase in virtual teams with a high average level of EI over time. We found a possible explanation for this unexpected finding related to the temporary nature of our teams. As mentioned above, computer-mediated communication has an important disadvantage, that is, team members’ tendency to behave in a more impersonal, hostile, and less empathic way within the team. These behaviors create an atmosphere of distrust, suspicion, and misunderstanding, hindering team members’ connection with each other. In this regard, it is possible that member’ efforts to emotionally manage teamwork focus especially on these negative affective states that arise in these first phases of virtual teamwork. It is possible that members working in teams with a relatively short lifespan, as in our teams (one month), prioritize managing members’ negative affective states, which could hinder team processes and the achievement of team goals. Future studies should examine the relationship between team EI composition and members’ positive affective states in long-term virtual teams or teams with a more extensive lifespan.

Another main objective of our study was to examine the impact of a team emotional management intervention on the relationship between team emotional intelligence composition and members’ well-being. We posited that an online intervention for virtual teams on the use of socio-emotional cues and emotional management strategies applied to text-based communication would moderate the impact of team EI composition. Our results showed that the TEM intervention moderated the relationships between team EI composition and members’ well-being (satisfaction with the team and negative affective states), buffering its influence. Thus, the influence of team EI composition was only significant in virtual teams with no intervention, becoming more important in this condition. Our findings support the efficacy of a TEM intervention for managing emotions in virtual teams where members communicate with each other through online written systems. This TEM intervention increased members’ competency in using verbal resources such as paralinguistic cues, management of timing, and emoticons to transmit socio-emotional information. Our TEM intervention provided virtual teams with a collective ability to manage expressions and displays of emotional cues during teamwork [56], thus improving interpersonal relationships [54]. Therefore, virtual teams that receive a team emotional management intervention are better at inducing and sustaining positive emotions among team members and building a positive team climate [51]. An unexpected result was that the team emotional management intervention reduced the stability of members’ negative affective experiences over time. Whereas there was a positive relationship between negative affective states at Time 1 and Time 3 in the control condition, this relationship disappeared in the intervention condition. Thus, effective emotional management within virtual teams contributes to reducing the initial levels of virtual team work-related tension, nervousness, and anxiety.

### 4.1. Theoretical and Practical Implications

One of the strengths of this study is the implementation of a longitudinal design that allowed us to examine our focal variables and their relationships across the virtual team’s lifespan, providing a richer understanding of the dynamic and changing emotional nature of virtual teams [32]. Additionally, we used multilevel modelling to analyze the antecedents of individual well-being at the team level and more accurately model virtual teams’ emotional phenomena [75].

Overall, the findings reported here have a number of implications for future theoretical developments in virtual team research. First, until recently, the virtual team literature had examined the role of teams’ demographic and personality composition as inputs or drivers of virtual teams’ effectiveness. Our results extend these findings by examining the impact of teams’ emotional intelligence composition. Second, much of the research on emotions in virtual teams has focused on the ways emotions are expressed through computer-mediated communication and the emotional impact of virtual teamwork (e.g., [11,46,76,77,78]). Nonetheless, few studies have analyzed how to promote a higher level of well-being within virtual teams. Gilson et al. [8], in their review on virtual team research in the past ten years, detected a need for knowledge about how members’ well-being is shaped in virtual teams from a multilevel perspective. Our findings show that emotional factors at the team level, such as team emotional intelligence composition, influence individual affective experiences. This result contributes to research on team composition as a structural property of groups [18]. Future studies should extend these findings and examine the role of individual differences between members of a virtual team. Literature on team diversity shows that individual differences in values, beliefs, attitudes, and predispositions have an impact on team members’ interactional behaviors and patterns and intergroup relationships [79,80]. Although studies of diversity effects in teams have primarily focused on group-level outcomes, research in other areas allows us to infer that diversity has an effect on emotional regulation and well-being (e.g., [81,82,83]). In addition, our results show that an online intervention to develop team competencies to manage teams’ emotional environment has an impact on team members’ well-being. Third, virtual teams are usually formed based on members’ task-related expertise and experience, without considering members’ emotional intelligence. Thus, our results show that an intervention based on developing team emotional management is a key element in reducing the potential negative influence when this emotional characteristic is not considered in the team design. Future studies should continue this research line and examine the ability of other collective emotional constructs, such as teams’ trait affect composition [18] or teams’ emotional openness [50], to improve this individual result in virtual teams. Scholars should also analyze the moderator role of the TEM intervention in influencing other predictors of members’ well-being in virtual teams, such as the team leader’s emotional intelligence (e.g., [41,78]) or the organizational emotional culture [20].

The increasing use of virtual teams presents a unique challenge in managing human resources [53]. Thus, our findings have several practical implications for organizations. First, from a staffing perspective, organizations that rely on virtual teams should consider integrating emotional intelligence into their current selection system. Moreover, organizations should invest resources in TEM training to increase virtual teams’ emotional competence and empower them to more effectively handle the emotional challenges created by the virtual environment. Organizations that offer TEM training may be able to increase awareness and management of the way emotions are expressed in virtual teams, as well as their impact on members’ well-being and, ultimately, virtual teams’ results. Online TEM training may also help organizations to deal with the potential detrimental effects of teams composed of members with scant emotional skills. TEM interventions provide team members with emotional strategies and resources that help them to express themselves, interact online, and understand their virtual colleagues, so that they can accomplish goals together even though they may never meet face-to-face. During the intervention, work team members receive a short training using audio-visual materials. Then, they practice the training contents through different exercises. For instance, several positive and negative situations that could be experienced during virtual work are presented to team members (e.g., “two team members are arguing while performing a group task”). For each situation, they are asked to indicate what emotional regulation strategies they would use to maintain a positive team climate and avoid a negative team climate. An on-line training, as proposed in this study, to improve teams’ affective management skills can easily be implemented. Organizations can create a web-based platform to train their employees anywhere and anytime, provided they have Internet access. Because these teams work with computer-mediated communication tools (e.g., Skype, Zoom, Blackboard Collaborate), the training can also be provided through this medium. Finally, given that it is a short online training program composed of two sessions (or blocks of content) lasting one hour each, team members can individually learn and practice at their own pace and convenience within the training time frame, and then agree with other teammates on a time frame for collective practice so that the training does not disrupt their work day.

### 4.2. Limitations

This study has some limitations. First, the data were based on self-reported measures. Hence, it might be argued that common method variance may have inflated the hypothesized relationships. Nonetheless, we minimized this problem by using aggregated data in our predictor [84] and measuring our focal constructs at different time points [85]. Future studies should consider other data sources, such as group discussion analyses, in order to examine members’ interactions and emotional expressions during teamwork. Second, individual satisfaction with the team and affective states could have been influenced by individual personality traits, typically neuroticism and extraversion [86]. Future studies should attempt to replicate our findings, controlling these personality characteristics. Third, our sample was composed of newly formed virtual teams, with a relatively short lifespan (one month). Thus, the temporary nature of our teams limits the generalization of the results to long-term teams. Fourth, our results have some limitations in terms of generalizability to organizational settings because they were obtained in a laboratory with a sample of students. Although there is ample agreement about the capacity of experimental studies to address applied problems in this field, and much of the research on virtual teams is conducted with students [8,87], future studies should try to replicate our results in real organizational contexts. Finally, the findings of the current study are only generalizable to virtual teams that utilize one type of computer-mediated communication, synchronous communication through electronic text-based chat in real time (i.e., instant messaging) [88]. Because multiple modalities of communication are available for virtual interactions (i.e., e-mail, videoconferencing), our results should be tested in teams that are communicating through different communication media.

## 5. Conclusions

In conclusion, very little is known about the role of team emotional composition in virtual teams, where, unlike in face-to-face teams, emotional expression cues are relatively limited. Although researchers have unequivocally started to demonstrate that team emotional composition plays a unique role in members’ well-being in face-to-face teams, to date, scholars have neglected to investigate the implications of these emotional constructs and their relationships in virtual teams. Our study identifies emotional intelligence as a key driver of virtual team members’ well-being, and it highlights the effectiveness of a team emotional management intervention to buffer its impact on virtual teams.

## Figures and Tables

**Table 1:** Descriptive statistics and correlations for the study variables in control condition: Teams without intervention.

Variable		*M*	*SD*	1	2	3	4	5	6	7	8
1	Team characteristics	-	-	-							
2	Satisfaction with the team S1	4.36	0.70	0.04	-						
3	Positive affective state S1	4.29	0.53	0.15 *	0.41 **	-					
4	Negative affective state S1	3.76	0.80	0.06	0.31 **	0.36 **	-				
5	Team EI composition	5.04	0.26	0.15 *	−0.05	0.14 *	0.11	-			
6	Satisfaction with the team S3	4.43	0.46	0.03	0.19 **	0.20 **	0.14 *	0.27 **	-		
7	Positive affective state S3	4.27	0.72	0.11	0.06	0.23 **	0.14 *	0.15 *	0.39 **	-	
8	Negative affective state S3	4.30	0.62	0.02	−0.05	−26 **	−24 **	0.30 **	0.35 **	0.57 **	-

Note. * *p* < 0.05; ** *p* < 0.01; two-tailed. EI = Emotional intelligence; S1 = Session 1; S3 = Session 3.

**Table 2:** Descriptive statistics and correlations for the study variables in experimental condition: Teams with intervention.

Variable		*M*	*SD*	1	2	3	4	5	6	7	8
1	Team characteristics	-	-	-							
2	Satisfaction with the team S1	4.37	0.70	−0.03	-						
3	Positive affective state S1	4.24	0.60	0.11	0.50 **	-					
4	Negative affective state S1	3.62	0.81	0.04	0.33 **	0.24 **	-				
5	Team EI composition	5.04	0.32	0.22 **	0.01	0.08	0.08	-			
6	Satisfaction with the team S3	4.49	0.52	0.28 **	0.27 **	0.19 **	0.01	0.28 **	-		
7	Positive affective state S3	4.45	0.64	0.20 **	0.31 **	0.44 **	0.21 **	0.17 *	0.36 **	-	
8	Negative affective state S3	4.37	0.68	0.13	0.13	0.19 **	0.06	0.06	0.31 **	0.52 **	-

Note. * *p* < 0.05; ** *p* < 0.01; two-tailed. EI = Emotional intelligence; S1 = Session 1; S3 = Session

**Table 3:** Multilevel estimates for models predicting satisfaction with the team in Session 3.

Variable	Null Model			Model 1			Model 2		
	Estimate	*SE*	*t*	Estimate	*SE*	*t*	Estimate	*SE*	*t*
Control condition: Teams without emotional management intervention									
Intercept	4.427	0.0643	68.791 **	3.3985	0.2933	11.58 **	0.8513	1.1990	0.710
Control Variables									
Satisfaction with the team S1 ^a^				0.2339	0.0645	3.627 **	0.2418	0.0641	3.772 **
TC ^b^				0.0089	0.0621	0.144	−0.0103	0.0604	−0.172
Predictor									
EI composition ^b^							0.5017	0.2297	2.183 *
−2 * log	409.20			403.66			400.14		
Difference of −2 * log				5.54 **			3.52*		
Experimental condition: Teams with emotional management intervention									
Intercept	4.492	0.0730	61.501 **	2.3456	0.3529	6.645 **	0.5675	1.035	0.548
Control Variables									
Satisfaction with the team S1 ^a^				0.4204	0.0731	5.747 **	0.4200	0.0727	5.777 **
TC ^b^				0.1532	0.0637	2.405 *	0.1279	0.0637	2.006 *
Predictor									
EI composition ^b^							0.3629	0.1994	1.819
−2 * log	473.57			445.72			443.85		
Difference of −2 * log				27.85 **			1.87		

^a^ Predictors at the individual level ^b^ Predictors at the team level Note. * *p* < 0.05; ** *p* < 0.01; two-tailed. EI= Emotional intelligence; TC = Team characteristics; S1 = Session 1.

**Table 4:** Multilevel estimates for models predicting positive affective states in Session 3.

Variable	Null Model			Model 1			Model 2		
	Estimate	*SE*	*t*	Estimate	*SE*	*t*	Estimate	*SE*	*t*
Control condition: Teams without emotional management intervention									
Intercept	4.2678	0.0561	75.987 **	3.0011	0.4036	7.435 **	1.5336	1.0248	1.496
Control Variables									
Positive affective state S1 ^a^				0.2814	0.0941	2.989 **	0.2661	0.0945	2.816 **
TC ^b^				0.0569	0.0522	1.091	0.0463	0.0520	0.891
Predictor									
EI composition ^b^							0.3058	0.1975	1.548
−2 * log	443.50			439.96			438.98		
Difference of −2 * log				3.54 *			0.98		
Experimental condition: Teams with emotional management intervention									
Intercept	4.4509	0.0503	88.379 **	2.3783	0.2853	8.333 **	1.3531	0.6794	1.992 *
Control Variables									
Positive affective state S1 ^a^				0.4434	0.0658	6.736 **	0.4388	0.0656	6.684 **
TC ^b^				0.0950	0.0410	2.314 *	0.0805	0.0413	1.949 *
Predictor									
EI composition ^b^							0.2128	0.1288	1.652
−2 * log	397.17			357.74			357.30		
Difference of −2 * log				39.43 **			0.44		

^a^ Predictors at the individual level ^b^ Predictors at the team level Note. * *p* < 0.05; ** *p* < 0.01; two-tailed. EI= Emotional intelligence; TC = Team characteristics; S1 = Session 1.

**Table 5:** Multilevel estimates for models predicting negative affective states in Session 3.

Variable	Null Model			Model 1			Model 2		
	Estimate	*SE*	*t*	Estimate	*SE*	*t*	Estimate	*SE*	*t*
Control condition: Teams without emotional management intervention									
Intercept	4.3043	0.0523	82.231 **	3.6591	0.2094	17.471 **	0.2652	0.8492	0.312
Control Variables									
Negative affective state S1 ^a^				0.1779	0.0579	3.363 **	0.1651	0.0512	3.221 **
TC ^b^				0.0240	0.0556	0.480	0.0497	0.0441	1.126
Predictor									
EI composition ^b^							−0.6870	0.1684	−4.080 **
−2 * log	380.43			377.48			364.57		
Difference of −2 * log				2.95			12.91 **		
Experimental condition: Teams with emotional management intervention									
Intercept	4.3717	0.0523	82.231 **	4.0400	0.2410	16.762 **	3.7353	0.9041	4.131 **
Control Variables									
Negative affective state S1 ^a^				0.0412	0.0579	0.712	0.0397	0.0581	0.685
TC ^b^				0.0901	0.0556	1.620	0.0858	0.0575	1.492
Predictor									
EI composition ^b^							−0.0632	0.1801	−0.351
−2 * log	423.19			427.81			429.28		
Difference of −2 * log				−4.62			−1.47		

^a^ Predictors at the individual level ^b^ Predictors at the team level. Note. ** *p* < 0.01; two-tailed. EI = Emotional intelligence; TC = Team characteristics; S1 = Session 1.

## Data Availability

The data presented in this study are available on request from the corresponding author.
